# Blueberry intake elevates post-exercise anti-inflammatory oxylipins: a randomized trial

**DOI:** 10.1038/s41598-023-39269-1

**Published:** 2023-07-24

**Authors:** David C. Nieman, Camila A. Sakaguchi, Ashraf M. Omar, Kierstin L. Davis, Cameron E. Shaffner, Renee C. Strauch, Mary Ann Lila, Qibin Zhang

**Affiliations:** 1 Human Performance Laboratory, Biology Department, Appalachian State University, North Carolina Research Campus, Kannapolis, NC USA; 2 UNCG Center for Translational Biomedical Research, University of North Carolina at Greensboro, North Carolina Research Campus, Kannapolis, NC USA; 3 Food Bioprocessing and Nutrition Sciences Department, Plants for Human Health Institute, North Carolina State University, North Carolina Research Campus, Kannapolis, NC USA

**Keywords:** Biochemistry, Immunology, Physiology, Systems biology

## Abstract

This study determined if 18 days of supplementation with blueberries (BL) compared to placebo (PL) could mitigate muscle soreness and damage and improve inflammation resolution in untrained adults (n = 49, ages 18–50 years) after engaging in a 90-min bout of “weekend warrior” eccentric exercise. The BL freeze dried supplement provided 1 cup of fresh blueberries per day equivalent with 805 mg/day total phenolics and 280 mg/day anthocyanins. Urine levels of eight BL gut-derived phenolics increased after 14- and 18-days supplementation with 83% higher concentrations in BL vs. PL (p < 0.001). The 90-min exercise bout caused significant muscle soreness and damage during 4d of recovery and a decrease in exercise performance with no significant differences between PL and BL. Plasma oxylipins were identified (n = 76) and grouped by fatty acid substrates and enzyme systems. Linoleic acid (LA) oxylipins generated from cytochrome P450 (CYP) (9,10-, 12,13-dihydroxy-9Z-octadecenoic acids) (diHOMEs) were lower in BL vs. PL (treatment effect, p = 0.051). A compositive variable of 9 plasma hydroxydocosahexaenoic acids (HDoHEs) generated from docosahexaenoic acid (DHA, 22:6) and lipoxygenase (LOX) was significantly higher in BL vs. PL (treatment effect, p = 0.008). The composite variable of plasma 14-HDoHE, 17-HDoHE, and the eicosapentaenoic acid (EPA)-derived oxylipin 18-hydroxyeicosapentaenoic acid (18-HEPE) (specialized pro-resolving lipid mediators, SPM, intermediates) was significantly higher in BL vs PL (treatment effect, p = 0.014). Pearson correlations showed positive relationships between post-exercise DHA-LOX HDoHEs and SPM intermediates with urine blueberry gut-derived phenolics (r = 0.324, p = 0.023, and r = 0.349, p = 0.015, respectively). These data indicate that 18d intake of 1 cup/day blueberries compared to PL was linked to a reduction in pro-inflammatory diHOMES and sustained elevations in DHA- and EPA-derived anti-inflammatory oxylipins in response to a 90-min bout of unaccustomed exercise by untrained adults.

## Introduction

Multiomics methods such as metabolomics and proteomics are used by sports nutrition investigators to capture the system-wide influence of nutritional interventions before and after stressful exercise workloads^1–8^. Lipid metabolites such as oxylipins have emerged as pivotal mediators of nutrition-exercise interactions^5,9^. Oxylipins are bioactive lipids that are produced via oxygenation of polyunsaturated fatty acids (PUFAs) such as linoleic acid (LA, 18:2), arachidonic acid (ARA, 20:4), eicosapentaenoic acid (EPA, 20:5), and docosahexaenoic acid (DHA, 22:6)^9–12^. Oxylipins are synthesized from cell membrane phospholipid PUFAs as they are released under regulation by phospholipase A2 (PLA2) in response to cell activation from various stress-related stimuli including exercise^13^. Cyclooxygenase (COX), lipoxygenase (LOX), and cytochrome P450 (CYP) enzyme systems metabolize the released PUFAs into oxylipins that act as autocrine and paracrine lipid mediators in numerous physiological processes. Depending on the metabolic context, oxylipins can function as beneficial signaling agents or mediators of inflammation, immune dysfunction, and disease^9–12,14^. A significant proportion of the physiological and immune system effects from n−6 and n−3 PUFAs are mediated through these oxidized lipid mediators^9,10,14^.

Emerging evidence indicates that lipid mediators are involved in initiating, mediating, and resolving exercise-induced muscle tissue injury, inflammation, and metabolic stress^15–24^. Previous studies indicate that increases in inflammatory cytokines and oxylipins following prolonged and intensive running or cycling can be countered through carbohydrate ingestion, with the largest effects seen for ARA-CYP-derived oxylipins^5,22^. A randomized clinical trial showed that 2-weeks ingestion of 1 cup blueberries per day increased plasma levels of gut-derived phenolics and countered increases in plasma levels of 10 proinflammatory oxylipins following a 75-km cycling bout^22^. A correlation analysis showed a negative relationship between this group of plasma blueberry metabolites with the 10 ARA-CYP oxylipins. These data demonstrated that oxylipins were sensitive to both exercise and nutritional influences.

The purpose of this study was to extend these findings from endurance athletes to relatively untrained individuals using plasma oxylipins as the primary outcome measure. This study determined if supplementation with blueberries (1 cup/day, 14 d before exercise and 4 days after) compared to placebo could mitigate muscle soreness and damage and improve inflammation resolution in response to an acute, 90-min bout of eccentric exercise. Common exercises that cause an eccentric muscle contraction include going down several flights of stairs, running downhill, lowering weights, stop-and-go sports like basketball and tennis, and the downward motion of squats, push-ups, or pull-ups. Eccentric muscle activity, especially in relatively untrained individuals, causes extensive and prolonged muscle damage and soreness^23^. Muscle damage from eccentric exercise induces a significant migration of immune cells and a corresponding inflammatory response^14,16–18^. Many individuals experience this type of muscle damage and soreness during unaccustomed exercise bouts on weekends or vacations.

## Results

**Figure 1:**
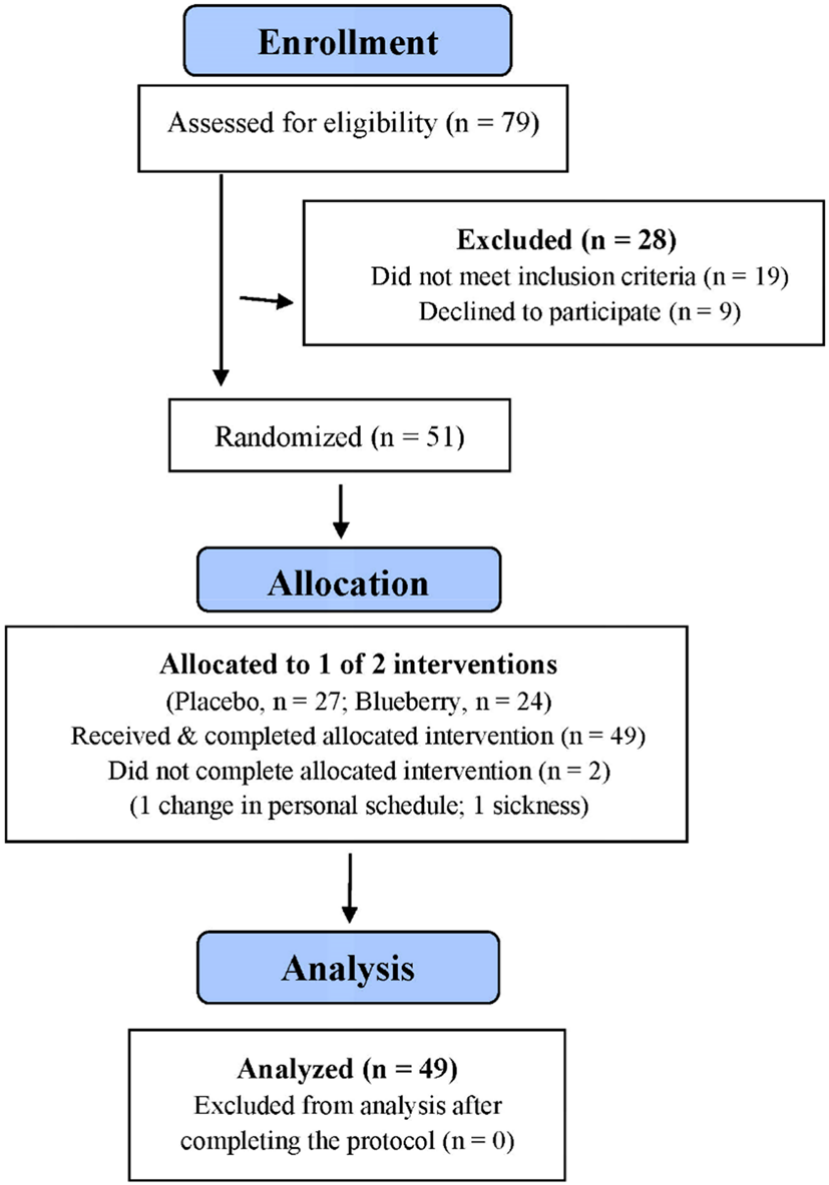
Subject flow diagram.

A total of 51 of 79 adults assessed for eligibility were randomized to blueberry and placebo groups (Fig. 1). Characteristics for the n = 49 study participants completing all aspects of the study protocol are summarized in Table 1. The blueberry (n = 23) and placebo (n = 26) groups did not differ in age, weight, height, body mass index (BMI), and body composition (body fat percentage). The proportion of males and females between groups did not differ (chi-square statistic = 0.023, p-value = 0.879). Male and female study participants did not differ significantly in key outcome responses across the seven measurement time points of this study including delayed onset of muscle soreness (DOMS), muscle damage (serum myoglobin), profile of mood states total mood disturbance (POMS TMD), and each of the oxylipin composite variables (all sex main effects, p > 0.05). Thus, male and female study participants were combined in the group comparisons.

**Table 1:** Comparison of subject characteristics between the placebo and blueberry groups. Mean ± SE.

	Placebo *(n* = *26) (12 M, 14F)*	Blueberry *(n* = *23) (12 M, 11F)*	T-test p-value
Age	39.0 ± 1.3	38.8 ± 1.3	0.942
Weight (kg)	72.1 ± 2.8	72.1 ± 2.8	0.984
Height (cm)	172 ± 1.9	172.3 ± 2.3	0.812
BMI (kg/m^2^)	24.3 ± 0.5	24.1 ± 0.5	0.839
% Body fat	22.0 ± 1.1	24.2 ± 1.8	0.294

*M* male, *F* female, *Kg* kilograms, *cm* centimeters, *BMI* body mass index.

Three-day food records collected at the beginning and end of the initial 14-day supplementation period were averaged to assess the background diets of the placebo and blueberry groups (not including the supplements). This analysis revealed no significant differences (all p > 0.05) in energy, carbohydrate, protein, fat, alcohol, micronutrient, and flavonoid intake between groups (data not shown). For all subjects combined, energy intake of the background diet averaged from the two 3-day food records was 2,097 ± 116 kcal/day (8.77 ± 0.49 MJ/day), with carbohydrate, protein, fat, and alcohol representing 42.2 ± 1.5%, 18.6 ± 0.8%, 38.7 ± 1.2%, and 1.9 ± 0.5%, respectively of total energy. Total flavonoid intake averaged 70.6 ± 25.8 mg/day.

The 90-min eccentric exercise bout had a negative effect on four of five performance outcomes including vertical jump height, bench press repetitions, 60-yard shuttle run time, and anaerobic power (peak and average watts) during the 30-s Wingate test (all time effects, p ≤ 0.004). The pattern of change in the five performance tests across the seven time points did not differ between the placebo and blueberry groups (all interaction effects, p > 0.05) (data not shown).

Muscle soreness (DOMS), POMS TMD scores, and muscle damage biomarkers (creatine kinase, myoglobin) are summarized in Table 2. The 90-min eccentric exercise bout increased muscle soreness and damage, with an increase in POMS TMD (all time effects, p ≤ 0.002). The patterns of change over time for all variables summarized in Table 2 did not differ between the placebo and blueberry groups (interaction effects, p > 0.05).

**Table 2:** Delayed onset of muscle soreness (DOMS), profile of mood states (POMS), and muscle damage data before and after 2-weeks supplementation, and immediately-post 90 min eccentric exercise and in an overnight fasted state during 4 days of recovery (PL = placebo, n = 26; BL = blueberry, n = 23) (means ± SE).

Variable	Group	Pre-Suppl	2-weeks Post-Suppl	0 h PostEx	1d PostEx	2d PostEx	3d PostEx	4d PostEx	Time; interaction *p* values
DOMS (1–10 scale)	PL	1.65 ± 0.19	1.52 ± 0.15	3.73 ± 0.40	5.69 ± 0.41	5.21 ± 0.41	3.94 ± 0.39	2.65 ± 0.36	< 0.001, 0.254
	BL	2.00 ± 0.26	1.78 ± 0.19	3.91 ± 0.46	6.17 ± 0.43	6.30 ± 0.45	4.67 ± 0.41	2.72 ± 0.32	
POMS Total mood disturbance	PL	93.2 ± 1.5	94.5 ± 1.6	98.0 ± 1.9	95.9 ± 1.8	95.4 ± 1.6	94.8 ± 2.0	95.2 ± 2.1	0.002, 0.172
	BL	92.0 ± 1.5	94.0 ± 2.0	98.0 ± 2.4	99.0 ± 2.1	97.7 ± 2.0	95.9 ± 2.1	93.7 ± 1.8	
Creatine kinase (Units/L)	PL	161 ± 23.3	124 ± 12.7	174 ± 18.2	355 ± 52.2	276 ± 43.0	275 ± 68.0	336 ± 109	< 0.001, 0.930
	BL	217 ± 54.4	174 ± 37.2	229 ± 40.8	575 ± 164	610 ± 262	742 ± 346	933 ± 453	
Serum myoglobin (ng/ml)	PL	34.7 ± 6.3	26.7 ± 1.3	129 ± 19.4	60.7 ± 14.1	36.8 ± 4.8	57.7 ± 14.1	54.3 ± 14.8	< 0.001, 0.797
	BL	31.5 ± 3.5	30.5 ± 3.2	145 ± 18.5	55.2 ± 13.9	73.4 ± 33.5	73.9 ± 29.8	87.0 ± 31.5	

Suppl = supplementation; PostEx = post-90 min eccentric exercise bout.

**Figure 2:**
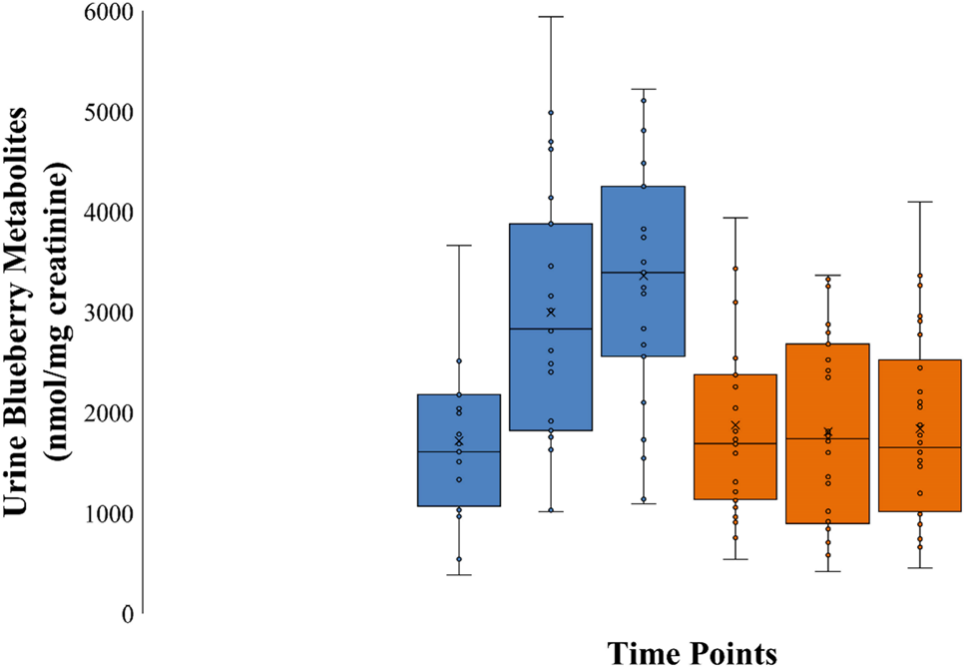
Sum of 8 urine blueberry gut-derived metabolites for the blueberry (blue) and placebo (orange) groups at three time points (pre-study, and after 14d and 18d supplementation. The pattern of change in this urine gut-derived phenolic composite variable was significantly different between groups (interaction effect, p < 0.001), with 83% higher concentrations in the blueberry compared to placebo group at the day 18 time point. This composite variable included hippuric acid, vanillic acid (4-hydroxy-3-methoxybenzoic acid), three ferulic acid-based metabolites (methoxy-cinnamic acid-O-glucoronate or sulfate forms), hydroxyphenyl-γ-valerolactone-*O*-sulfate, methoxy-catechol sulfate, and methoxyphenylacetic acid-O-sulfate.

Pre- and post-2 weeks, and 18-days supplementation 24 h urine volumes for the placebo (1691 ± 178, 1757 ± 164, 1923 ± 158 ml) and blueberry (1691 ± 134, 1798 ± 179, 2065 ± 163 ml) groups were not significantly different (all times points, p > 0.50). Of 31 targeted phenolic metabolites detected in the urine samples, eight with group contrast differences at the end of the study (all p < 0.10) were combined into a composite variable. This composite variable included hippuric acid, vanillic acid (4-hydroxy-3-methoxybenzoic acid), three ferulic acid-based metabolites (methoxy-cinnamic acid-O-glucoronate or sulfate forms), hydroxyphenyl-γ-valerolactone-O-sulfate, methoxy-catechol sulfate, and methoxyphenylacetic acid-O-sulfate. The pattern of change in this urine gut-derived phenolic composite variable was significantly different between groups (interaction effect, p < 0.001), with 83% higher concentrations in the blueberry compared to placebo group (Fig. 2).

**Figure 3:**
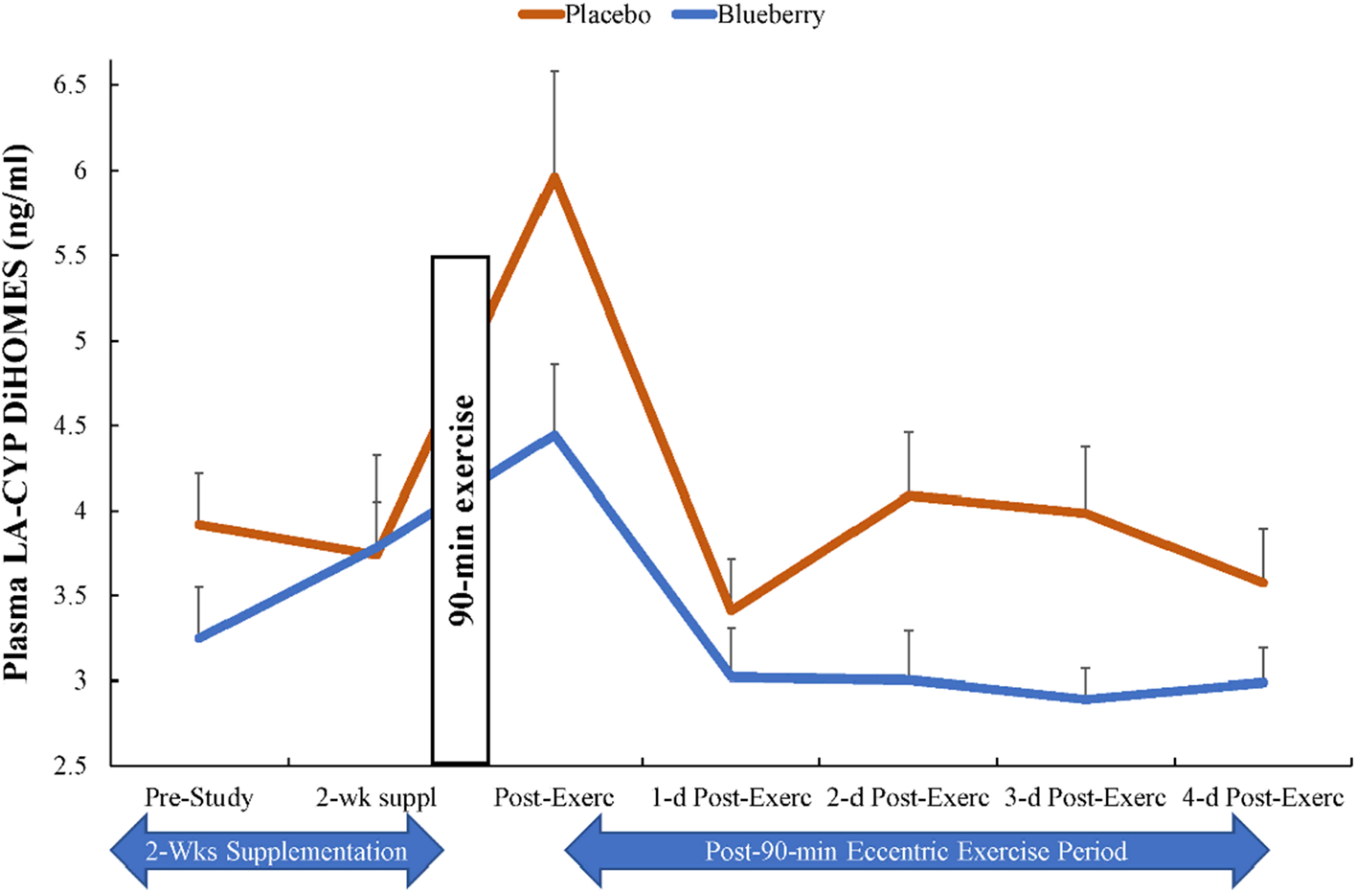
The pattern of change over time in linoleic acid (LA) oxylipins generated from CYP (9,10-DiHOME and 12,13-DiHOME) tended to be lower in the blueberry group (time effect, p < 0.001; interaction effect, p = 0.062; supplement group main effect, p = 0.051). Blueberry and placebo ingestion continued during 4 days of post-exercise recovery.

**Figure 4:**
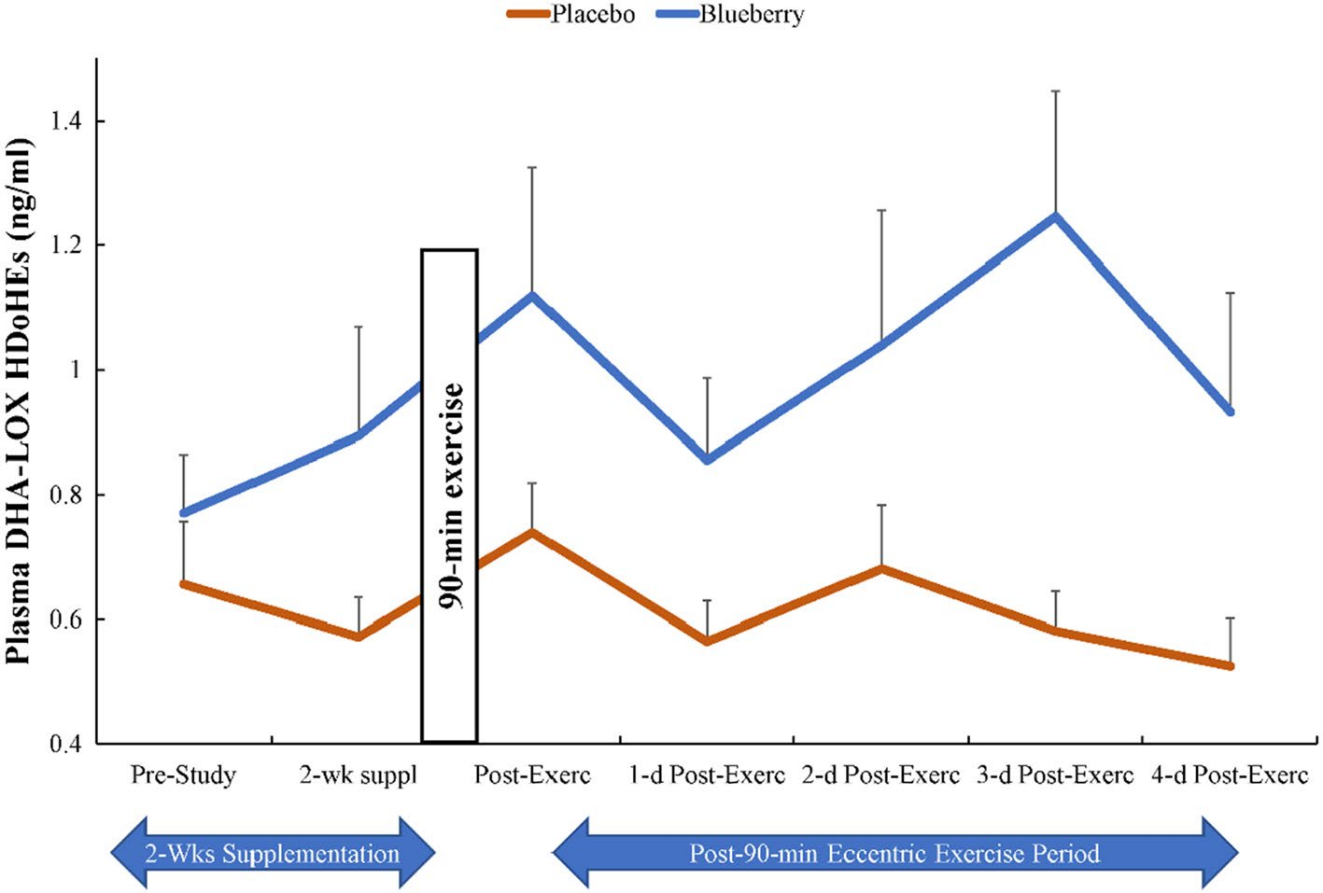
Plasma DHA-LOX HDoHEs were higher in the blueberry compared to placebo group (treatment effect, p = 0.008; interaction effect, p = 0.385). DHA-LOX: 4-,7-,8-,10-,11-,13-,14-,16-,17-HDoHEs. Blueberry and placebo ingestion continued during 4 days of post-exercise recovery.

**Figure 5:**
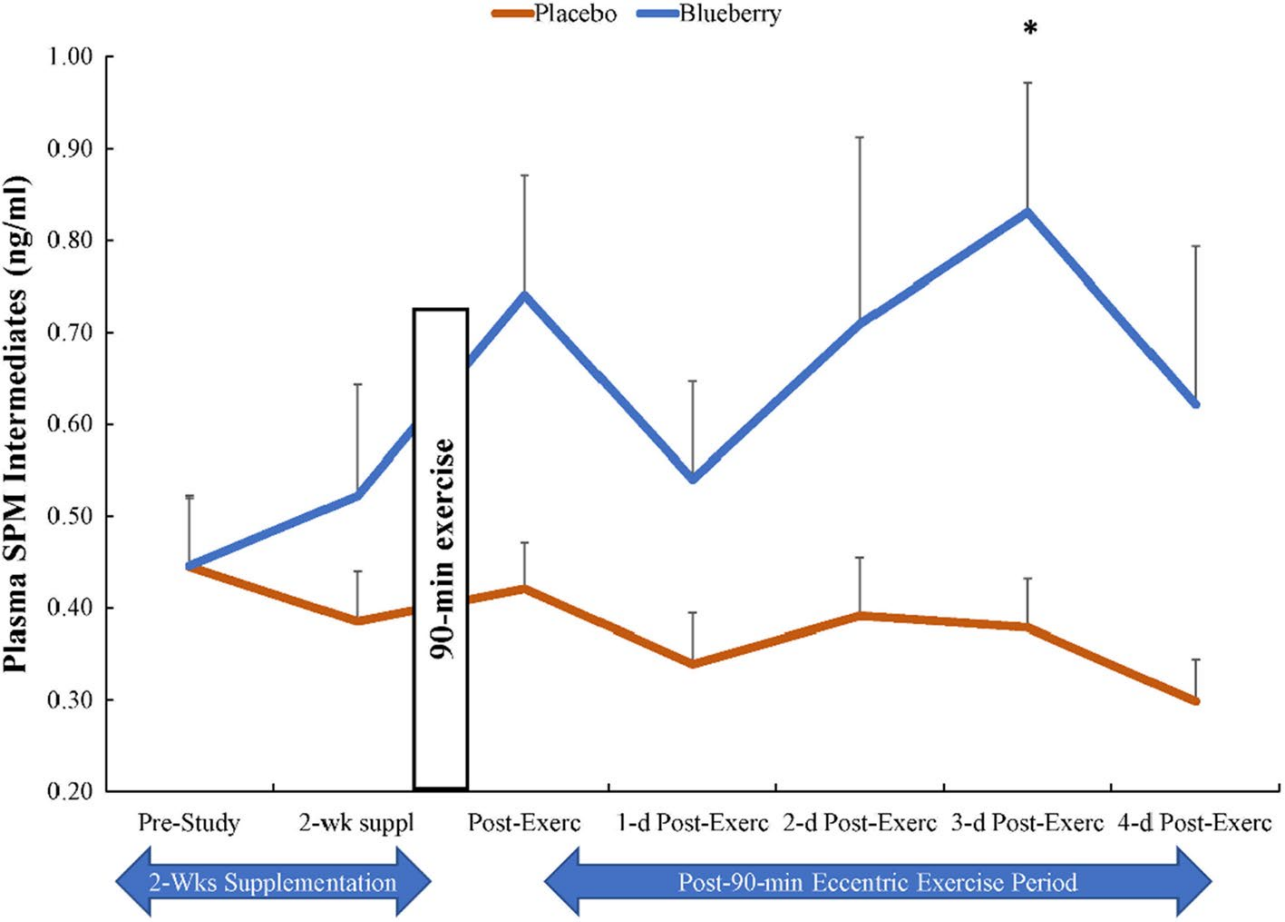
SPM intermediates (specialized pro-resolving lipid mediators) were higher in the blueberry compared to placebo group (treatment effect, p = 0.014; interaction effect, p = 0.192). SPM = the sum of plasma 14-HDoHE, 17-HDoHE, and 18-HEPE. Blueberry and placebo ingestion continued during 4 days of post-exercise recovery.

The 90-min eccentric exercise bout caused immediate post-exercise increases in plasma concentrations of ARA, EPA, and DHA fatty acids with no differences between groups (time effects, all p ≤ 0.001) (data not shown). A total of 76 oxylipins were quantified in the study plasma samples (Supplemental Table S1). For all subjects combined, 9 of 76 oxylipins increased significantly immediately post-exercise (0.80- to 1.53-fold) including 9- and 13- hydroxy-octadecadienoic acids (HODEs), 9,10-, 12,13- dihydroxy-9Z-octadecenoic acids (diHOMEs), 5,6-, 14,15-dihydroxy-eicosatrienoic acid (diHETrEs), 9-hydroxy-octadecatrienoic acid (9-HOTrE), and 11-,15-hydroxy-eicosatetraenoic acid (HETEs). Detected oxylipins were grouped according to relevant fatty acid substrates and enzyme systems and these composite variables were analyzed for time and supplement treatment main effects and interaction effects. 9-,13-HODEs produced from LA-LOX increased significantly post-exercise (time effect, p < 0.001), with no group or interaction differences (Table 3). Small but significant time effects were found for ARA-CYP and ARA-LOX (p < 0.05), with no group or interaction differences (Table 3). No significant time, group, or interaction effects were found for EPA-LOX and ARA-COX (Table 3). The pattern of change over time in linoleic acid (LA) oxylipins generated from CYP (9,10-, 12,13-diHOMEs) tended to be lower in the blueberry group (time effect, p < 0.001; interaction effect, p = 0.062; supplement group main effect, p = 0.051) (Fig. 3). The plasma concentration of a composite variable of nine DHA-LOX-derived hydroxydocosahexaenoic acids (HDoHEs) was significantly higher in the blueberry compared to the placebo group (time effect, p = 0.034; treatment effect, p = 0.008; interaction effect, p = 0.385) (Fig. 4). The plasma concentration of the composite variable of plasma 14-HDoHE, 17-HDoHE, and the EPA-derived oxylipin 18-hydroxyeicosapentaenoic acid (18-HEPE) (specialized pro-resolving lipid mediators, SPM, intermediates) was significantly higher in the blueberry compared to placebo group (time effect, p = 0.050; treatment effect, p = 0.014; interaction effect, p = 0.192) (Fig. 5). Only two SPMs were detected in the plasma samples and these included DHA-derived protectin D1 and 7(R) maresin-1. These were summed into an SPM composite variable, and the group comparison showed no differences (time effect, p = 0.654, supplement effect, p = 0.645) (Table 3).

**Table 3:** The plasma oxylipin dataset was grouped according to substrate (linoleic acid, LA; eicosapentaenoic acid, EPA; docosahexaenoic acid DHA) and enzyme systems (cytochrome P-450, CYP; lipoxogenase, LOX).

Plasma oxylipin group (ng/ml)			Group	Pre-Suppl		2-weeks Post-Suppl		0 h PostEx		1d PostEx	2d PostEx		3d PostEx		4d PostEx		Time; Interaction; Group effects, *p* values	
LA-LOX			PL	5.90 ± 0.70		6.00 ± 0.72		8.79 ± 0.78		4.95 ± 0.44	6.26 ± 0.60		5.56 ± 0.39		5.15 ± 0.42		< 0.001, 0.266, 0.208	
			BL	4.61 ± 0.45		5.79 ± 0.95		7.80 ± 0.96		4.48 ± 0.40	4.58 ± 0.40		4.98 ± 0.39		5.13 ± 0.51			
ARA-CYP			PL	6.66 ± 0.43		6.65 ± 0.51		6.90 ± 0.61		7.07 ± 0.67	8.09 ± 0.81		7.47 ± 0.60		7.43 ± 0.66		0.025, 0.267, 0.708	
			BL	6.58 ± 0.34		6.55 ± 0.41		7.55 ± 0.36		7.10 ± 0.47	7.03 ± 0.46		6.69 ± 0.39		7.03 ± 0.34			
ARA-LOX			PL	2.04 ± 0.19		1.86 ± 0.11		2.37 ± 0.19		1.81 ± 0.13	2.10 ± 0.21		1.83 ± 0.12		1.78 ± 0.12		0.031, 0.402, 0.240	
			BL	2.06 ± 0.13		2.27 ± 0.19		2.40 ± 0.29		1.99 ± 0.14	2.15 ± 0.18		2.39 ± 0.20		2.00 ± 0.12			
ARA-COX			PL	1.74 ± 0.17		2.06 ± 0.34		2.19 ± 0.23		1.82 ± 0.24	1.87 ± 0.14		1.89 ± 0.21		1.95 ± 0.20		0.228, 0.691, 0.763	
			BL	1.87 ± 0.14		1.72 ± 0.15		2.10 ± 0.19		1.75 ± 0.13	1.72 ± 0.17		1.72 ± 0.16		2.24 ± 0.31			
EPA-LOX			PL	0.26 ± 0.04		0.23 ± 0.02		0.25 ± 0.02		0.19 ± 0.03	0.23 ± 0.02		0.19 ± 0.03		0.20 ± 0.02		0.832, 0.457, 0.597	
			BL	0.21 ± 0.03		0.22 ± 0.03		0.23 ± 0.04		0.24 ± 0.03	0.25 ± 0.03		0.22 ± 0.04		0.26 ± 0.03			
SPMs			PL	0.17 ± 0.02		0.20 ± 0.03		0.21 ± 0.03		0.16 ± 0.02	0.19 ± 0.02		0.19 ± 0.02		0.21 ± 0.03		0.654, 0.085, 0.645	
			BL	0.23 ± 0.03		0.14 ± 0.02		0.18 ± 0.02		0.21 ± 0.02	0.19 ± 0.02		0.25 ± 0.03		0.19 ± 0.02			

Data are summarized before and after 2-weeks supplementation, and immediately-post 90 min eccentric exercise and in an overnight fasted state during 4 days of recovery (PL = placebo, n = 26; BL = blueberry, n = 23) (means ± SE).

Suppl = supplementation; PostEx = post-90 min eccentric exercise bout.

LA-LOX: 9-,13-HODEs.

ARA-CYP: 16-HETE, 20coohAA, 5,6-, 8,9-, 11,12-, 14,15-DiHETrEs.

ARA-LOX: 5-,8-,9-,11-,12-,15-HETEs; 5-,15-oxoETE; tetranor 12-HETE; LTB4; 6S-LXA4; 15-R-LXA4.

ARA-COX: 12-HHTrE, TXB2, PGA2, PGB2, PGD2, PGE2, PGF2α, PGJ2, 15 k PGE2, 15 k PGD2, dhkPGD2, dhk PGF2α, bicyclo PGE2.

EPA-LOX: 5-,8-,9-,11-,12-,15-HEPEs.

SPMs: protectin D1 and 7(R) maresin-1.

*ARA* arachidonic acid, *DHA* docosahexaenoic acid, *EPA* eicosapentaenoic acid, *LA* linoleic acid, *COX* cyclooxygenase, *CYP* cytochrome P450, *LOX* lipoxygenase, *20cooh AA* 20-carboxy-arachidonic acid, *DiHDPA* dihydroxy-docosapentaenoic acid, *DiHETE* dihydroxy-eicosatetraenoic acid, *DiHETrE* dihydroxy-eicosatrienoic acid, *DiHOME* dihydroxy-9Z-octadecenoic acid, *HDoHE* hydroxy-docosahexaenoic acid, *HEPE* hydroxy-eicosapentaenoic acid, *HETE* hydroxy-eicosatetraenoic acid, *HHTrE* hydroxyheptadecatrienoic acid, *HODE* hydroxy-octadecadienoic acid, *LX* lipoxin, *LT* leukotriene, *PG* prostaglandin, *TX* thromboxane.

**Figure 6:**
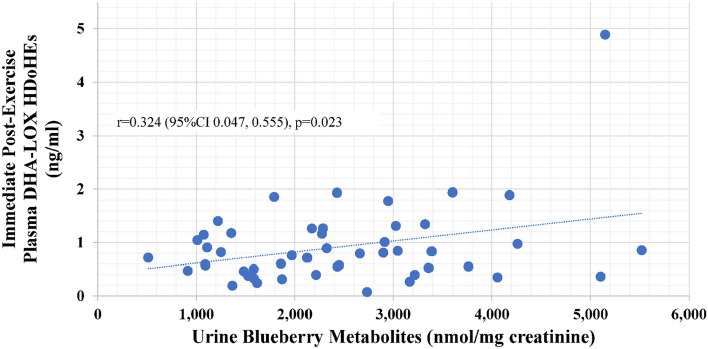
Positive correlation (r = 0.324, p = 0.023) between urine blueberry metabolites (average of 14d and 18d supplementation 24-h urine samples) and immediate post-exercise plasma concentrations for DHA-LOX HDoHEs.

**Figure 7:**
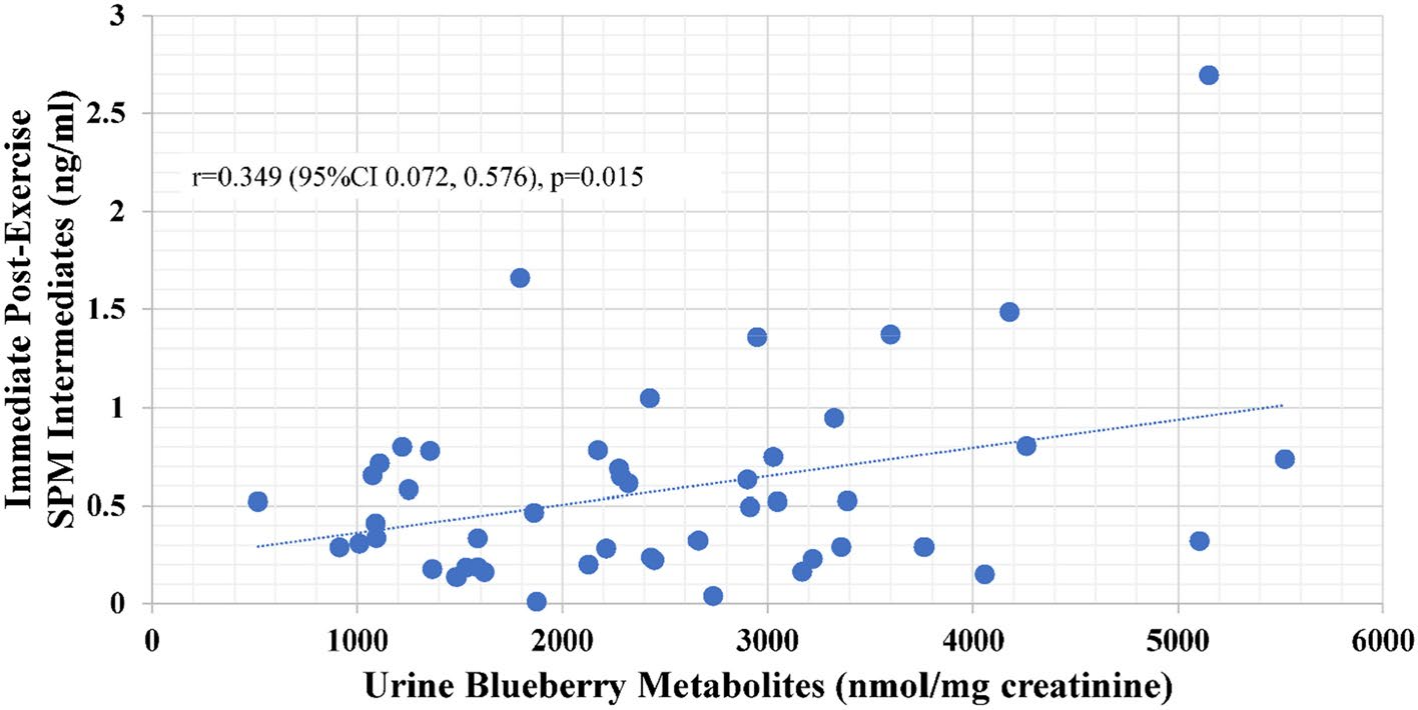
Positive correlation (r = 0.349, p = 0.015) between urine blueberry metabolites (average of 14d and 18d supplementation 24-h urine samples) and immediate post-exercise plasma concentrations for SPM intermediates (14-HDoHE, 17-HDoHE, 18-HEPE). *SPM* specialized pro-resolving lipid mediators.

Pearson correlations were calculated for the relationships between specific oxylipin composite variables (LA-LOX, DHA-LOX HDoHEs, and SPM intermediates with the average of days 14 and 18 for the blueberry gut-derived phenolics concentrations in the 24 h urine collections. Significant positive relationships were found for DHA LOX HDoHEs (r = 0.324, p = 0.023) and SPM intermediates (r = 0.349, p = 0.015) at the immediate post-exercise time point (but not any other time points) (Figs. 6, 7).

## Discussion

This randomized, placebo-controlled clinical trial showed that blueberry ingestion during an 18-d period decreased plasma concentrations of pro-inflammatory diHOMEs and increased anti-inflammatory DHA- and EPA-generated HDoHE and SPM-intermediate oxylipins during the 4-day recovery period after an acute 90-min bout of unaccustomed exercise in untrained adults. Blueberry intake did not alter the post-exercise increase in muscle soreness and damage, or the downturn in exercise test performance.

Relatively few acute and chronic exercise-based studies have been conducted using oxylipins as a primary outcome measure^5,15–24^. In general, the acute post-exercise increase in plasma levels of oxylipins is more diverse and greater with prolonged and intensive cardiorespiratory exercise (e.g., 2–3 h of cycling and running) than with resistance-based exercise bouts^5,16–18,22–24^. After 2–3 h of intensive cycling or running, plasma oxylipins peak between 0- and 1.5 h post-exercise and then fall to near-pre-exercise levels by 5 h post-exercise^22^. In two studies conducted by our research group (including the current study) using a 90-min eccentric exercise protocol, fold increases of 9 to 13 oxylipins varied from 0.8 to 2.8 primarily for the most abundant oxidized lipids generated from LA (9,10-, 12,13-diHOMEs, and 9-,13-HODEs)^23^. In contrast, after 75-km cycling, plasma concentrations of 64 of 67 identified oxylipins were elevated with an average 12-fold increase^22^.

Nutrition supplements including arachidonic fatty acids, sports beverages, bananas, blueberries, almonds, aronia citrus juice, and astaxanthin been investigated for their potential influence on the oxylipin response to varying exercise modes and workloads^5,15,18,22–24^. Carbohydrate intake from sports beverages or bananas consumed during exercise (0.8 g/kg each hour of exercise) exerted strong effects in reducing plasma concentrations of ARA-CYP proinflammatory oxylipins after exhausting endurance exercise^5,22^. Blueberries (1 cup/day, 15 days) in a previous study from our research group also decreased post-75 km cycling plasma levels of ARA-CYP oxylipins, but to a lesser extent than carbohydrates^22^. The current study showed that 1 cup/d blueberries was efficacious in decreasing plasma concentrations of LA-CYP derived diHOMES while promoting a sustained elevation in plasma concentrations of DHA- and EPA-generated oxylipins known for their upstream anti-inflammatory effects during several days of recovery from an acute bout of eccentric-based exercise^14^. This is a novel finding that may only apply to this physiological stress context in untrained adults. During recovery from 75-km cycling in trained endurance athletes, for example, blueberry intake mitigated exercise-induced increases in pro-inflammatory oxylipins but did not elevate anti-inflammatory omega 3-generated oxylipins^22^. In a similar design to the current study, we recently reported that 4-weeks intake of 57 g/day almonds was related to a significant post-exercise increase in 12,13-diHOME and a decrease in 9,10-diHOME, with no significant effects on other oxylipins^23^. This disparate diHOME response to eccentric-based exercise may have in part been related to the unique nutrient and polyphenol profile of almonds^23^. Previous studies indicate that diHOMEs are elevated in various disease and injury conditions, but this response and the net physiological impact can vary depending on the overall stress context^25^. Taken together, the results from these sports nutrition studies indicate that the oxylipin response differs depending on the exercise mode and workload, and the type of nutritional intervention^12^. Our research group used the same research design and exercise challenge as in a previous study with almonds^23^, but the oxylipin response to blueberry ingestion differed significantly underscoring the importance of nutrition in modulating the generation of lipid mediators.

Underlying mechanisms are still being explored but limited data suggest that increases in gut-derived phenolics and changes in the gut microbiome from added polyphenol intake may influence oxylipin production through effects on related enzyme systems including cPLA2, CYP, sEH, LOX, and COX^26–28^. Human data are lacking but the available data suggest modulations of the gut microbiota with blueberry and anthocyanin ingestion^28^. If true, the change in gut microbiota diversity may amplify the generation of gut-derived phenolics linked to increased blueberry intake over time. The positive correlations between concentrations of urine blueberry gut-derived phenolics and post-exercise plasma DHA-LOX HDoHEs and SPM intermediates support this potential relationship^26,27^. These correlation data were significant only at the immediate post-exercise time point and explained only a small proportion of the variance. Previous research from our group has shown that walking or running augments the acute release of gut-derived phenolics from the gut to the circulation^8^. Although undetermined, measurement of blueberry gut-derived phenolics in the plasma compared to prior day urine concentrations may have provided stronger statistical linkages with post-exercise plasma oxylipin concentrations.

In this study, blueberry intake was related to higher post-exercise plasma levels of SPM intermediate oxylipins but not the actual SPMs. This finding is comparable to results from a study of EPA and DHA supplementation (10 weeks) with systemically inflamed adults^11^. The investigators hypothesized that increased EPA and DHA intake would cause a replacement of n−6 ARA with these n−3 fatty acids in cell membrane phospholipids and thereby improve the supply of DHA and EPA substrates and the generation of SPMs^11^. EPA and DHA supplementation was linked to increases in SPM intermediates but without changes in plasma SPMs including resolvins, maresins, or protectins. The oxylipin measurement system used in the current study included standards for most of the important SPMs and was sensitive enough to measure their concentrations in plasma if present, but only two were detected (protectin D1 and 7(R) maresin-1)^5,22–24^. SPMs have a well-defined role in inflammation resolution but these oxylipins may not accumulate in the plasma or muscle tissue during and following stressful exercise workloads^5,16^. A study of 14 untrained males also reported inconsistently low or undetectable levels of SPMs in a series of muscle biopsies collected after an acute exercise bout of maximal concentric and eccentric knee extensions^16^.

This investigation and others have shown that diet-based interventions can influence the oxylipin response to exercise stress without effects on muscle soreness and damage or exercise performance^22,23^. Several review papers have concluded that increased intake of blueberries, anthocyanins, and polyphenols in general may have a small, variable, or null effect on mitigating exercise-induced muscle soreness, damage, and dysfunction^29–36^. Changes in muscle damage biomarkers such as creatine kinase and myoglobin following eccentric exercise are highly variable between individuals as confirmed in this study. The underlying mechanisms explaining this variance is poorly understood but may be due to part to single nucleotide polymorphisms (SNPs)^37^. Interindividual variability in biomarker responses to eccentric exercise-induced muscle damage due to SNPs may limit the efficacy of nutritional interventions. Lipid mediators generated from DHA and EPA have been hypothesized to enhance muscle regeneration by regulating the inflammatory response to muscle injury, but definitive evidence in exercise-based studies is lacking^20,38,39^. Daily intraperitoneal injection of DHA-derived resolvin D1 (RvD1) in aged mice suppressed inflammatory cytokine expression and improved recovery of muscle function and was advanced as a promising treatment of muscular injuries and pain in the elderly^39^. The long-term effect of regular blueberry ingestion on muscle soreness and damage repair in exercising adults remains to be determined, but our data do not support a positive effect during a 4-day recovery period after one acute muscle-damaging exercise bout. A limitation of this study is that higher numbers of subjects in each group with measurements of SNPs would be necessary to have sufficient statistical power and information to determine if blueberry ingestion influences muscle damage.

In conclusion, this study demonstrated that a proper balance between pro-inflammatory and pre-resolution pathways can be supported by a nutritional intervention within an exercise stress context. Inflammation resolution is a complex process that involves tissue rebuilding, regulation of excessive neutrophil migration and oxidative burst activity, stimulation of local macrophages, clearance of cell debris, and the proper balance of both oxylipin and cytokine production^14^. An 18-day supplementation period with 1 cup/d equivalent of blueberries enhanced the inflammation resolution process following a “weekend warrior” exercise session by mitigating increases in LA-CYP diHOMEs and enhancing plasma concentrations of anti-inflammatory DHA-LOX HDoHE and SPM-intermediate oxylipins.

## Methods

### Study participants

Study participants included healthy, non-smoking male and female adults 18 to 50 years of age with a body mass index (BMI) less than 30 kg/m^2^. Other inclusion criteria were similar to those from a recent study^23^ and included no recent history of engagement in regular resistance training (less than 3 sessions per week), and a willingness to avoid the use of protein supplements, large-dose vitamin/mineral and herbal supplements, and anti-inflammatory medications during the project. A total of 79 participants were assessed for eligibility and 51 were randomized to placebo and blueberry groups. Of these, 49 completed all aspects of the study protocol (Fig. 1). The minimum number of study participants for adequate study power was estimated (https://clincalc.com/stats/samplesize.aspx) using 9,10-diHOME data (a key outcome) from a previous study conducted by our research group using a very similar design^23^. This analysis showed that a minimum of 14 participants per group would provide 90% power to detect a difference at an alpha level of 0.05 using two-sided t-tests. Participants voluntarily signed the informed consent, and procedures were approved by the Appalachian State University Human Subjects Institutional Review Board (IRB), Federal Wide Assurance (FWA) number: FWA00027456. Notice of IRB approval by full board review was granted by the IRB (#20-0213) on 15/9/2020. The research was performed in accordance with relevant guidelines and regulations, and informed consent was obtained from all study participants. Trial Registration: ClinicalTrials.gov, U.S. National Institutes of Health, identifier: NCT05184855, 11/1/2022.

### Study design

This study employed a randomized parallel group, placebo-controlled, double-blinded design with a 2-week supplementation period, 90-min eccentric exercise bout challenge, and seven lab visits at the Appalachian State University Human Performance Laboratory (HPL) at the North Carolina Research Campus, Kannapolis, NC. The parallel group design was utilized as in previous studies because of the "repeat exercise bout" effect (i.e., post-exercise muscle damage and inflammation are reduced when the same bout is repeated within days and weeks of the first bout)^23^. The primary outcome measure was plasma oxylipins, and secondary measures included urine blueberry metabolites and muscle damage markers. Investigators and study participants were blinded to treatments until after the study and sample analysis were completed. Subject recruitment was initiated in January of 2022, with data collection and sample analysis completed by November of 2022.

Study participants were recruited by the research manager (C.S.) and randomized to the blueberry and placebo groups using random.org (1:1 allocation). The 2-week supplementation period was based on previous studies with blueberries showing bioactive effects within this length of time^6,22^. Participants in the study ingested 25 g/day of freeze-dried blueberries (1 cup fresh blueberries equivalent) or 25 g/d placebo powder. The powders were coded in packets, and double-blinded procedures were employed throughout the study period. Table 4 provides a summary of the blueberry and placebo supplements (25 g portions). The blueberry supplement was a freeze-dried blend (50/50) of Tifblue and Rubel highbush blueberries. The blueberry and placebo supplements had similar carbohydrate and sugar profiles, with the blueberry supplement providing 805 mg/day total phenolics and 280 mg/day total anthocyanins (analysis provided by the study sponsor, U.S. Highbush Blueberry Council, Folsom, CA). The blueberry and placebo supplements were of similar color and taste, and participants consumed the powders by mixing them in water, fruit juices, yogurts, or other food/beverage products.

**Table 4:** Comparison between the blueberry and placebo supplements.

Nutrient	Blueberry (25 g)	Placebo (25 g)
Phenolics (mg)	805	0
Anthocyanins (mg)	280	0
Calories (kcal)	99	91
Carbohydrate (g)	23.3	22.5
Sugars (g)	14.6	17.1
Fructose (g)	6.95	8.9
Glucose (g)	7.7	8.1
Dietary fiber (g)	5.6	0.24

Blood samples were collected in overnight-fasted study participants before and after 2-weeks supplementation. Additional blood samples were collected immediately post-exercise, and then each morning (overnight fasted state) during four days of recovery. Three 24-h urine samples were collected (total urine output) before and after the 2-week supplementation period, and then the day before the final blood draw (day 18). Ascorbic acid (300 mg) and boric acid (10 g) were added to the 24-h urine containers to help stabilize the phenolic molecules. After each of the blood draws (each overnight fasted except immediately post-exercise), participants completed the abbreviated Profile of Mood States (POMS) questionnaire using procedures described in an earlier study in our lab^40^, provided a muscle soreness rating using a 1–10 scale (DOMS)^23^, and completed the muscle function testing protocol. Participants recorded all food and beverage intake for three days before and at the end of the 2-week supplementation period to assess the background diet. Macro- and micro-nutrient, and flavonoid intake was assessed using the Food Processor dietary analysis software system with an adapted nutrient database to capture total flavonoid intake as described previously^41^ (Version 11.1, ESHA Research, Salem, OR, USA).

Study participants signed the consent form and were given a complete orientation to the study protocol during the first lab visit. The orientation included an introduction of the exercises included in the eccentric exercise bout and the muscle function tests. Study participants practiced the exercises and function tests during the orientation period. During the second lab visit (overnight fasted, pre-supplementation), study participants provided a blood sample, turned in the 24-h urine collection bottle, and provided responses to POMS and DOMS questionnaires. Height and body weight were assessed, with body composition measured using the BodPod system (Cosmed, Rome, Italy). Participants then performed five muscle function tests as described previously^23^: vertical jump, bench press, leg-back strength, 60-yard shuttle run test, and anaerobic power through the 30-s Wingate test. Briefly, in the vertical jump test, participants jumped as high as possible with one hand, and tapped the measuring device (Vertec vertical jump apparatus, Questtek Corp, Northridge, CA, USA). In the bench press to exhaustion, participants laid down supine on the bench, and bench pressed a weighted bar equal to 50% (females) or 75% (males) of their body weight as many times as possible at a rate of 30 lifts per minute. Leg/lower back isometric lifting strength was assessed with a dynamometer (Lafayette Instruments, Lafayette, IN, USA). The Lode cycle ergometer (Lode B.V., Groningen, Netherlands) was used for the 30-sWingate cycling test. The workload was adjusted to the body mass of the subject (0.7 Newton meters per kilogram), and participants cycled at maximal speed for 30 s. The peak and total wattage power output were recorded and adjusted to body mass. In the 60-yard shuttle run test, participants sprinted progressing distances back and forth (5 yard, 10 yards, 15 yards), with the total time recorded in seconds. Study participants were then provided a 2-weeks supply of blueberry or placebo packets and told to return in 2 weeks for the eccentric muscle exercise bout. Supplementation compliance was monitored with regular email messages and the return of supplement wrappers.

Participants returned for their third lab visit after the 2-week supplementation period in an overnight fasted, turned in 3-day food records and 24-h urine bottles, gave responses to DOMS and POMS questionnaires, and provided a blood sample. Blueberry and placebo supplements (half doses) were ingested followed by muscle function testing. As described previously, participants then engaged in a 90-min eccentric exercise bout that consisted of 17 different exercises, most with 2 to 3 sets and 30–60 s of rest between sets^23,42^. Participants ingested another supplement half dose later that day and then returned at 7:00 am in an overnight fasted state four days in a row after the eccentric exercise bout, and provided blood samples, POMS and DOMS ratings, and repeated the five physical fitness tests. Blueberry and placebo supplementation was continued during this 4-day recovery period.

### Sample analysis

Blood samples were collected in serum separation tubes (SST) and ethylenediaminetetraacetic acid (EDTA) containing blood collection tubes. SST were spun at 2300 rpm for 15 min after being allowed to clot for 15 min. Serum creatine kinase and myoglobin were analyzed each day samples were collected using Labcorp services (Burlington, NC). Plasma aliquots were prepared from ethylenediaminetetraacetic acid (EDTA) containing blood collection tubes and stored in a − 80 °C freezer until analysis for the oxylipins after the study was completed.

#### Plasma oxylipins

Plasma arachidonic acid (ARA), eicosapentaenoic acid (EPA), docosahexaenoic acid (DHA), and oxylipins were analyzed using a liquid chromatography-multiple reaction monitoring mass spectrometry (LC-MRM-MS) method as fully described elsewhere^43^. Resultant data files were processed with Skyline^44^, and the auto-integrated peaks were inspected manually. Concentrations of each oxylipin were determined from calibration curves of each analyte, which were constructed by normalizing to the selected deuterated internal standards followed by linear regression with 1/x weighting (Supplemental Table S1). The coefficient of variation for the quality control standards was < 15% as reported in the method development paper^43^.

#### Urine phenolic metabolite analysis

Urine creatinine was quantified using an optical method relying on the Jaffe reaction in a 96-well format [modified from^45^]. Creatinine concentration was utilized for pre-acquisition normalization of samples to standardize the analytical conditions. Phenolic metabolites were purified from urine samples by SPE (Strata™-X 33 µm Polymeric Reversed Phase, 10 mg/well, 96-Well Plates, Phenomenex, Torrance, CA) using a method adapted from Nieman et al.^8^. UPLC–ESI–TOF-MRM analysis was performed using a Waters Xevo G2-XS QTOF mass spectrometer (Waters Corporation) coupled to an ACQUITY I-Class UPLC (Waters Corporation, Milford, MA). A sample volume of 1.5 µL was separated on a Kinetex 2.6 µm PFP 100 Å 100 × 2.1 mm LC column (Phenomenex) maintained at 37 °C. A binary gradient using 0.1% formic acid in water (Mobile phase A) and 0.1% formic acid in acetonitrile (mobile phase B), from 2% B to 90% B over 25 min and flow rate gradient ranging from 0.55 mL/min to 0.75 mL/min was utilized for separation^8^. The TOF-MRM method was created in negative mode for 9 standard metabolites and 28 putative metabolites; these were selected by interrogation of the pooled sample, investigation of proposed pathways utilizing mammalian and microbial enzymes for the conversion of anthocyanins to phenolic acids and related compounds^46^, as well as by analysis of results from previous clinical studies examining metabolites in human fluids after blueberry consumption^22^. The instrument was tuned and calibrated before each analysis run, phloridzin was utilized as an internal standard and pooled samples were monitored throughout the study for quality control. Metabolite peaks were analyzed using TargetLynx software (Waters Corporation).

### Statistical analysis

The data are expressed as mean ± SE and were analyzed using the generalized linear model (GLM), repeated measures ANOVA module in SPSS (IBM SPSS Statistics, Version 28.0, IBM Corp, Armonk, NY, USA). Between group study participant characteristics were contrasted using independent t-tests (Table 1). The statistical model utilized the between-subjects approach: 2 (groups) × 7 (time points) repeated measures ANOVA and provided time (i.e., the collective effect of the eccentric exercise bout), treatment (i.e., the collective effect of the entire treatment period including the 2-week supplement period and the 4-day exercise and recovery period), and interaction effects (i.e., whether the data pattern over time differed between groups). If the treatment or interaction effect was significant (p ≤ 0.05), then post-hoc analyses were conducted using Student’s t-tests comparing time point contrasts between groups. The treatment effect was relied on because the study employed three different phases including the initial 14-d supplementation period, the exercise challenge day, and a 4-d recovery period. An alpha level of p ≤ 0.007 was used after Bonferroni correction for 7 multiple tests. Pearson correlation coefficients and the corresponding p-values were calculated between targeted oxylipin composite variable and the summed group of urine metabolites.

## Supplementary Information

Supplementary Information.

## Data Availability

The datasets generated during and/or analyzed during the current study are available from the corresponding author on reasonable request.
